# Physiological oxygen tension reduces hepatocyte dedifferentiation in *in vitro* culture

**DOI:** 10.1038/s41598-017-06433-3

**Published:** 2017-07-19

**Authors:** Ren Guo, Xinxiu Xu, Yuting Lu, Xin Xie

**Affiliations:** 1 CAS Key Laboratory of Receptor Research, the National Center for Drug Screening, Shanghai Institute of Materia Medica, Chinese Academy of Sciences, Shanghai, 201203 China; 2 University of Chinese Academy of Sciences, No.19A Yuquan Road, Beijing, 100049 China; 3 School of Life Science and Technology, ShanghaiTech University, Shanghai, China; 4 Shanghai Key Laboratory of Signaling and Disease Research, Laboratory of Receptor-based Bio-medicine, School of Life Sciences and Technology, Tongji University, Shanghai, 200092 China

## Abstract

Primary hepatocytes cultured *in vitro* are a powerful tool to study the functions of hepatocytes and to evaluate the metabolism and toxicity of new drugs. However, *in vitro* culture of hepatocytes has proven to be very difficult. Ordinary culture conditions lead to dedifferentiation of hepatocytes, resulting in rapid change in cell morphology and significant reduction in specific cell functions. In the current study, we show that hepatocyte dedifferentiation is a rapid process under 21% O_2_ conditions. Hepatocytes cultured in 21% O_2_ undergo epithelial-to-mesenchymal transition (EMT), obtain fibroblast-like morphology, and show decreased hepatic functions. In contrast, 5% O_2_ is very effective in maintaining the epithelial morphology and many functions of the primary hepatocytes cultured *in vitro* for up to five days. These functions include albumin production, glycogen storage, LDL-uptake and CYP450-mediated drug metabolism. Furthermore, we find that 5% O_2_ can relieve the production of reactive oxygen species (ROS) and decrease the level of DNA damage in primary cultured hepatocytes. In addition, we also show that blocking the ERK and GSK-3β pathways can inhibit the dedifferentiation of hepatocytes to a certain extent. Lowering the oxygen tension in cell culture is easily achievable, we believe it could be combined with other methods, such as the use of small molecule cocktails and 3D culture, to maintain proliferation and functions of primary hepatocytes *in vitro*.

## Introduction

The liver is one of the most important and complex organs in the body as it plays a central role in the metabolic homeostasis^1^. It is also the main detoxifying organ, which removes wastes and xenobiotics by metabolic conversion and biliary excretion^2^. The liver is comprised of a multitude of cell types. However, the hepatic parenchymal cell, also known as the hepatocyte, is the main cell type and executes most functions of the liver^3^. Primary cultured human or rodent hepatocytes are great tools to study the development and functions of the liver, such as the investigation of transcription factors involved in liver development^4, 5^, and the exploration of molecular mechanisms involved in the liver’s response to cytokines and growth factors during liver regeneration^6, 7^. Primary hepatocytes are also important tools for the study of drug metabolism, toxicity and transporter functions^8, 9^. When isolated and cultured correctly, they can reserve the metabolizing enzymes and transporter activity at a physiological level and be regulated via the cellular processes which occur *in vivo* ^8^.

However, many basic functions of hepatocytes, such as bile canaliculi formation, bile secretion, polarity and metabolic activities (including detoxification by Cyps and other drug-metabolizing enzymes), are rapidly reduced during *in vitro* culture^10^. The loss of hepatocytes’ polarity and functions is called hepatocyte dedifferentiation. A number of pathways, including transforming growth factor (TGF)-β, sonic hedgehog (Hh), Wnt, Notch, epithelial growth factor (EGF), fibroblast growth factor (FGF), platelet-derived growth factor (PDGF), hypoxia induced factor (HIF), and many others^11^, have been reported to play a role in hepatocyte dedifferentiation in *in vitro* culture. It has also been reported that hepatocytes dedifferentiation is a consequence of overactive Ras/MEK/ERK signaling^12^. To resolve the dedifferentiation problem and preserve the functions of primary hepatocytes in *in vitro* culture, scientists have optimized the culture conditions by modifying the media components, adding small molecule compounds^13–15^, culturing in bioreactors or 3D systems^16, 17^, or co-culturing the hepatocytes with non-parenchymal cells^18^. As previously reported, a multiwall co-culture system based on elastomeric stencils for human liver cells and mouse 3T3-J fibroblasts could be optimized to form proper microscale architecture and could maintain the phenotypic functions of hepatocytes for several weeks^19^.

The 3D culture of hepatocytes or co-culture of hepatocytes with other hepatic cell types intends to mimic the *in vivo* microenvironment of the liver. One important fact that should be considered is the oxygen tension in the *in vivo* environment. In the human liver, hepatocytes reside in a physiologically low oxygen environment; the partial pressure of oxygen (*p*O_2_) in liver is (5.4 ± 0.7)% (40.6 ± 5.4 mm Hg)^20^, which is much lower than the *in vitro* condition (~21%, 160 mm Hg). In cell culture, oxygen tension has a great influence on cell fate. Notably, low O_2_ tension promotes the survival of neural crest cells and hematopoietic stem cells, enhances the generation of induced mouse pluripotent stem cells, and prevents differentiation of human ESCs^21, 22^. Recently, low O_2_ tension has been reported to enhance the generation of lung progenitor cells, definitive endoderm cells, and distal lung cells from mouse pluripotent stem cells, and enhances the generation of retinal progenitor cells from human pluripotent stem cells^23–25^.

In this study, we sought to evaluate the function of physiological oxygen tension in *in vitro* culture of primary hepatocytes, and found that 5% O_2_ reduces the epithelial-mesenchymal transition (EMT) of the hepatocytes, and helps to preserve the functions of hepatocytes.

## Result

### Physiological oxygen level helps to maintain the properties of primary hepatocytes in culture

**Figure 1:**
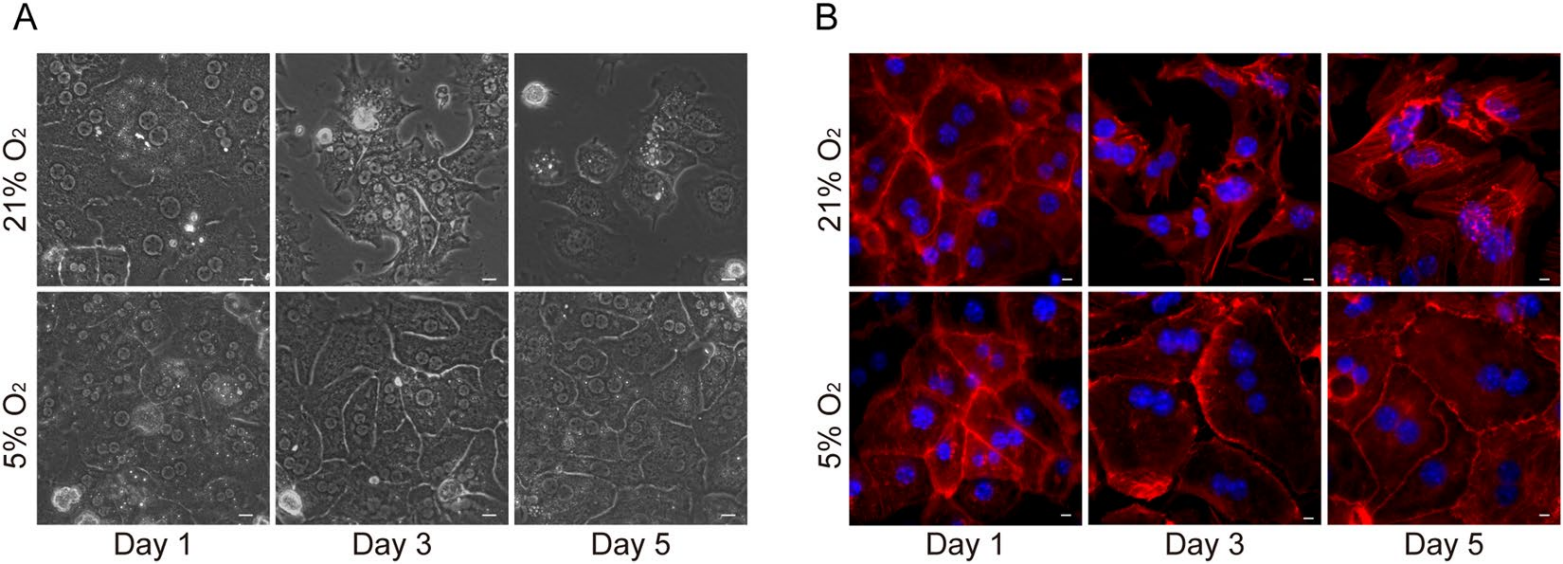
Physiological oxygen level helps maintain the endothelial phenotype of primary hepatocytes. (**A**) Morphology of primary hepatocytes cultured in 21% or 5% O_2_. Scale bar: 50 μM. (**B**) Immunofluorescence staining of F-actin (red) in primary hepatocytes cultured in 21% or 5% O_2._ Nuclei were stained with Hoechst. Scale bar: 10 μm.

Mouse primary hepatocytes cultured in 21% O_2_ underwent rapid morphological changes. At day 1 in culture, these cells exhibited typical endothelial phenotype. However, at day 3 and 5, these hepatocytes spread out and started to lose cell-cell contacts and finally obtained the fibroblast morphology (Fig. 1A). In contrast, hepatocytes cultured in 5% O_2_ maintained the epithelial phenotype for 5 days (Fig. 1A). Staining of F-actin confirmed this observation. At day 1 in both culture conditions, actin fibers were mainly located on the inner side of the cell membrane and formed a belt like structure around the cells. However, when cultured in 21% O_2_, the actin cytoskeletons started to re-structure and form the stress fiber as typically seen in fibroblasts (Fig. 1B). In contrast, the actin structure remained unchanged in the cells cultured in 5% O_2_ (Fig. 1B).

**Figure 2:**
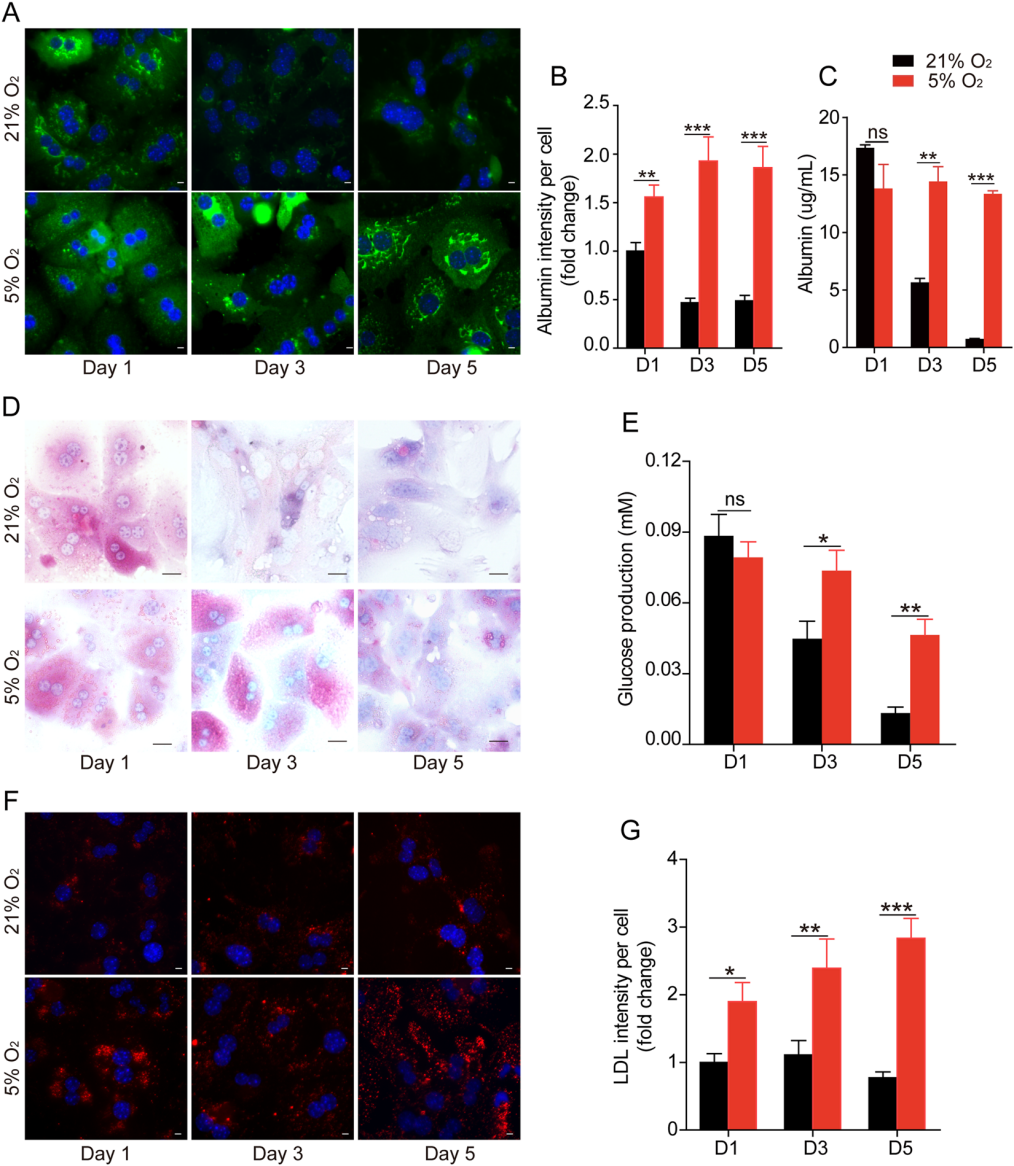
Physiological oxygen level preserves primary hepatocyte functions. (**A**) Immunofluorescence staining of albumin (green) in hepatocytes cultured in 21% or 5% O_2_. Nuclei were stained with Hoechst. Scale bar: 10 μm. (**B**) Statistical data of the intensity of albumin staining in (**A**). Data are shown as fold change relative to the 21% O_2_ group in day 1. Data are Means ± SEM (six random fields) in a representative experiment. (**C**) ELISA analysis of the secreted albumin levels in culture media of primary hepatocytes cultured in 21% or 5% O_2_. Data are Means ± SEM (n = 3). (**D**) PAS staining of the glycogen storage of primary hepatocytes cultured in 21% or 5% O_2_. Scale bar: 50 μm. (**E**) Glucose production of primary hepatocytes cultured in 21% or 5% O_2_. (**F**) Representative immunofluorescence images of Dil-Ac-LDL uptake (red) in primary hepatocytes cultured in different conditions. Scale bar: 10 μm. (**G**) Statistical data of the mean intensity of LDL per cell as shown in (**D**). Data are shown as fold change relative to the 21% oxygen group in day 1. Data are Means ± SEM (seven random fields) in a representative experiment. *p < 0.05, **p < 0.01, ***p < 0.001.

The ability to produce albumin is an important index of hepatocyte function. The intracellular staining of albumin decreased rapidly in hepatocytes cultured in 21% O_2_ (Fig. 2A,B), and the secreted albumin in hepatic culture was also significantly reduced (Fig. 2C). But in 5% O_2_, both the intracellular and secreted albumin remained unchanged (Fig. 2A–C). Glycogen storage and glucose output are also important functions of hepatocytes. Periodic acid-schiff (PAS) staining revealed that 21% O_2_ culture condition led to a rapid decrease of glycogen stores in primary hepatocytes (Fig. 2D). And gluconeogenesis from these cells was also greatly reduced (Fig. 2E). In contrast, hepatocytes cultured in 5% O_2_ remained PAS positive at day 5, and the glucose output ability was maintained (Fig. 2D,E). LDL-uptake ability was also evaluated in hepatocytes cultured in those two conditions. As expected, hepatocytes cultured in 5% O_2_ displayed a significantly higher ability to intake ac-LDL as compared to the cells cultured in 21% O_2_ (Fig. 2F,G).

### Hepatocytes cultured in physiological oxygen level maintain the expression and function of CYP450

**Figure 3:**
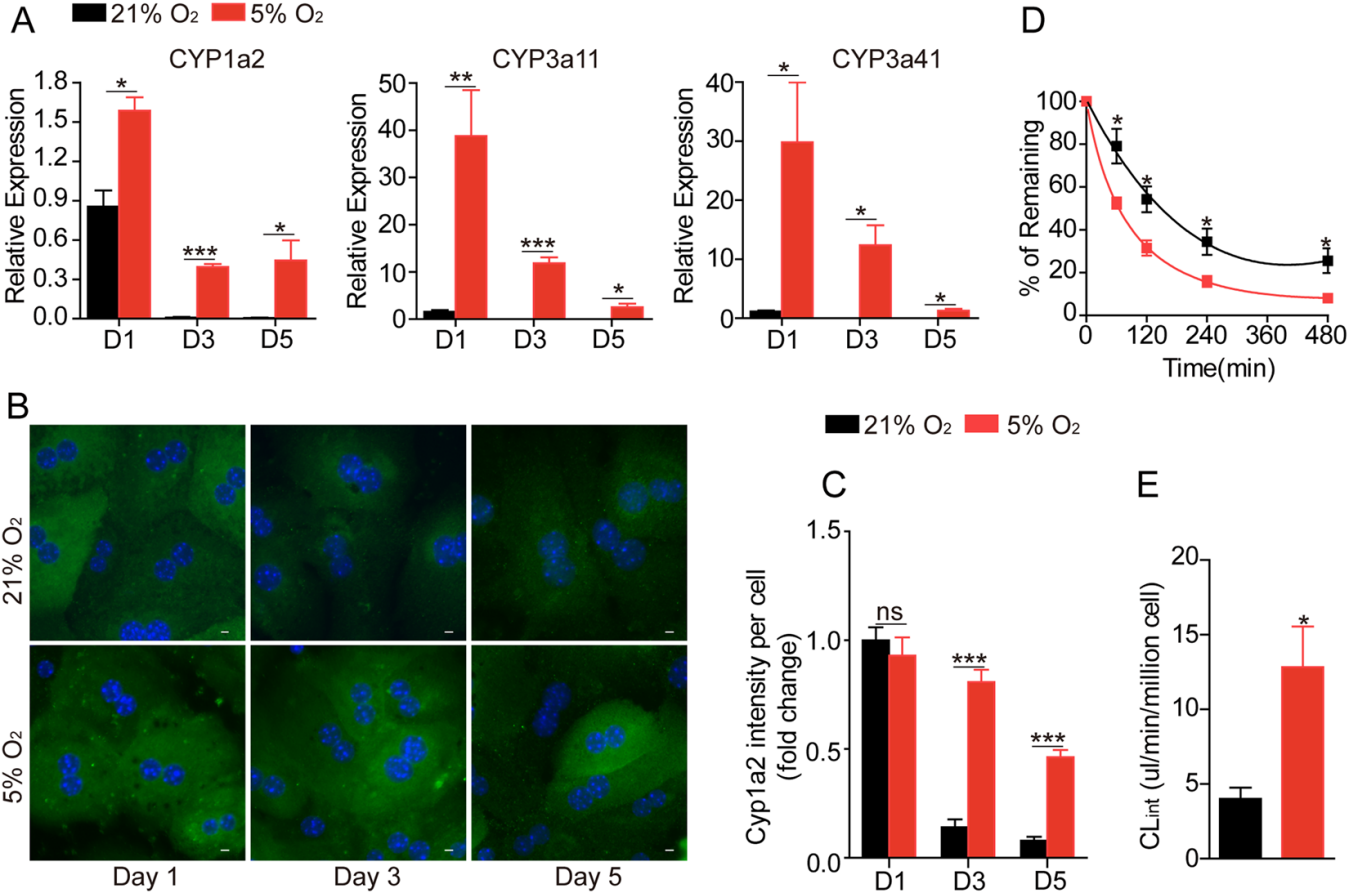
Physiological oxygen level maintains the expression and function of cytochrome P450 enzymes in primary hepatocytes. (**A**) Quantitative RT-PCR measurement of Cyp1a2, Cyp3a11 and Cyp3a41 in hepatocytes cultured in 21% or 5% O_2_. (**B**) Representative immunofluorescence images of Cyp1a2 in primary hepatocytes cultured in different conditions. Scale bar: 10 μm. (**C**) Statistical data of the mean intensity of Cyp1a2 per cell as shown in (**B**). Data are shown as fold change relative to the 21% oxygen group in day 1. Data are Means ± SEM (seven random fields) in a representative experiment. (**D**) Evaluation of the metabolic stability of phenacetin (200 nM) in primary hepatocytes cultured in 21% or 5% O_2_ for 3 days (n = 3). (**E**) The *in vitro* intrinsic clearance (CL_int_) of phenacetin was determined from the metabolic stability study presented in Fig. 3D. n = 3, *p < 0.05, **p < 0.01, ***p < 0.001.

The liver is the most important organ for drug metabolism. Approximately 60% of the marketed drugs are cleared by hepatic CYP450-mediated metabolism. *In vitro* culture led to rapid reduction in mRNA levels of CYP1a2, 3a11 and 3a41 in hepatocytes in both the 21% O_2_ and 5% O_2_ conditions. However, the reduction was significantly faster in hepatocytes cultured in 21% O_2_. At day 3, the mRNAs of these enzymes were almost undetectable in cells cultured in 21% O_2_, but in cells cultured in 5% O_2_, the expression of these enzymes was still detectable at day 5 (Fig. 3A). Immunofluorescent staining also confirmed a significant decrease of Cyp1a2 protein in hepatocytes cultured in 21% O_2_ (Fig. 3B,C).

Hepatocytes cultured in 21% O_2_ or 5% O_2_ for 3 days were then subjected to an *in vitro* drug metabolism assay. The *in vitro* clearance of phenacetin, a pain-relieving and fever-reducing drug and a substrate of CYP1a2, was tested. As demonstrated in Fig. 3D, hepatocytes cultured in 5% O_2_ displayed a higher clearance ability for phenacetin. After 480 min incubation, more than 90% phenacetin was cleared by hepatocytes cultured in 5% O_2_, but in the 21% O_2_ group, only about 60% of the phenacetin was cleared. The *in vitro* intrinsic clearance for phenacetin is significantly higher in hepatocytes cultured in 5% O_2_ than 21% O_2_ (13.33 vs 4.09 ul/min/million cells, respectively) (Fig. 3E).

### Physiological oxygen level blocks EMT involved in dedifferentiation of primary hepatocytes

**Figure 4:**
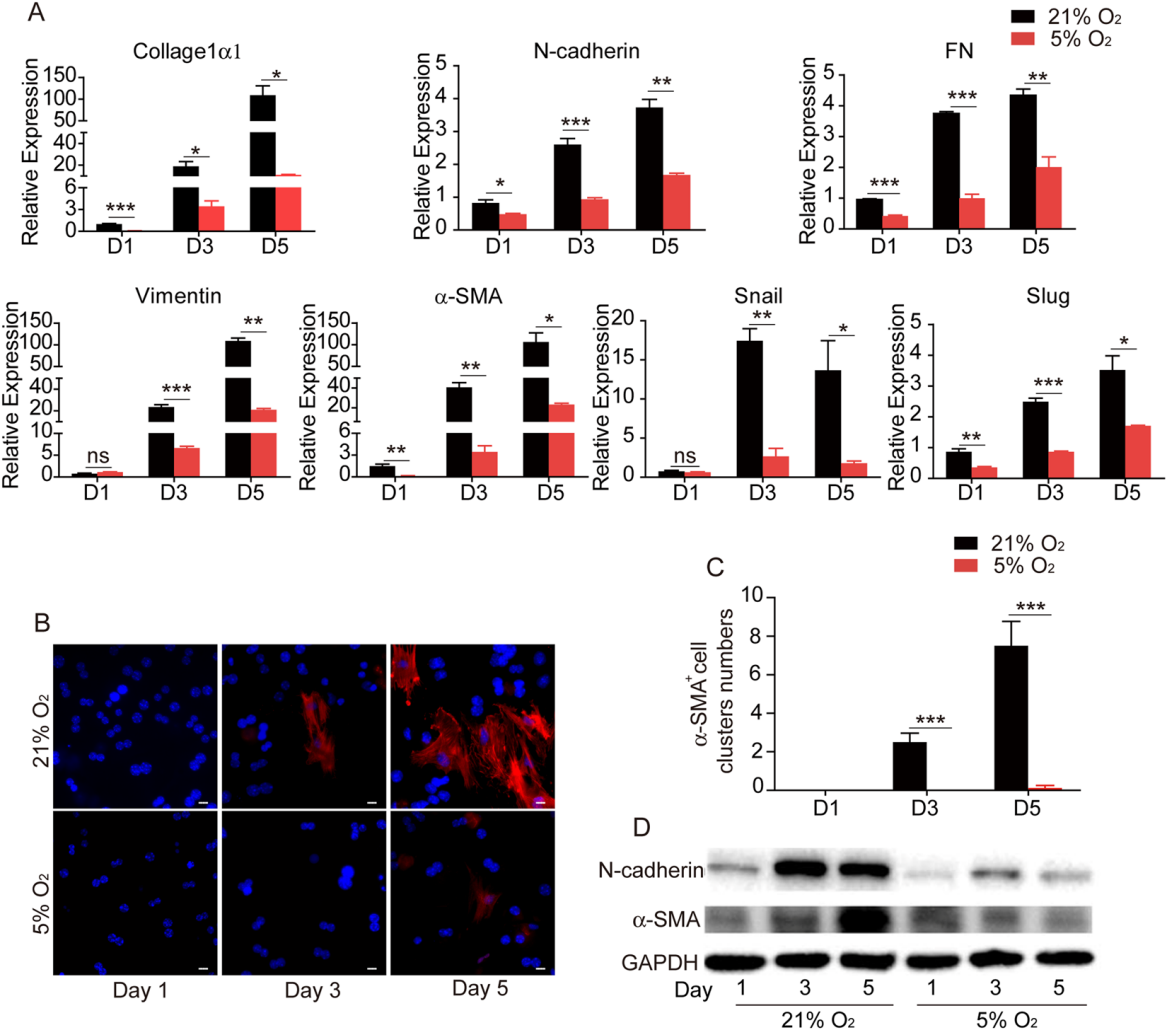
Physiological oxygen level suppresses EMT in the *in vitro* culture of primary hepatocytes. (**A**) Quantitative RT-PCR analysis of mesenchymal marker *collage1α1, N-cadherin, FN, Vimentin, α-SMA, snail*, and *slug* in hepatocytes cultured in 21% or 5% O_2_. Data are Means ± SEM (n = 3). (**B**) Immunofluorescence staining of α-SMA in hepatocytes cultured in 21% or 5% O_2_. Nuclei were stained with Hoechst. Scale bar: 20 μm. (**C**) Statistical data of the number of α-SMA^+^ clusters as shown in (**B**). Data are Means ± SEM (eight random fields) in a representative experiment. *p < 0.05, **p < 0.01, ***p < 0.001. (**D**) Western blot analysis of N-cadherin and α-SMA in hepatocytes cultured in 21% or 5% O_2_. GAPDH: loading control.

As we demonstrated above, mice primary hepatocytes cultured in 21% O_2_ quickly lost the typical endothelial phenotype and obtained the fibroblast morphology, while 5% O_2_ culture condition prevented such morphological changes (Fig. 1). The morphological changes involved in hepatocyte dedifferentiation were accompanied by the dramatic upregulation of mesenchymal markers, including *Collage1α1, N-cadherin, FN, Vimentin, α-SMA, Snail*, and *Slug*, further indicating the occurrence of epithelial-mesenchymal transition (EMT) during long-term culture of primary hepatocytes in 21% O_2_ condition. In contrast, 5% O_2_ significantly prevented the upregulation of all the mesenchymal-related genes (Fig. 4A). Immunofluorescent staining also confirmed the significant increase of *α*-SMA^+^ cells in hepatocytes cultured in 21% O_2_ for 3 and 5 days. However, in 5% O_2_ condition, very few *α*-SMA^+^ cells were detected (Fig. 4B and C). Western blot analysis also revealed the protein levels of N-cadherin and *α*-SMA were increased in 21% O_2_ but not in 5% O_2_ culture condition (Fig. 4D).

### Low oxygen leads to less oxidative stress and DNA damage

**Figure 5:**
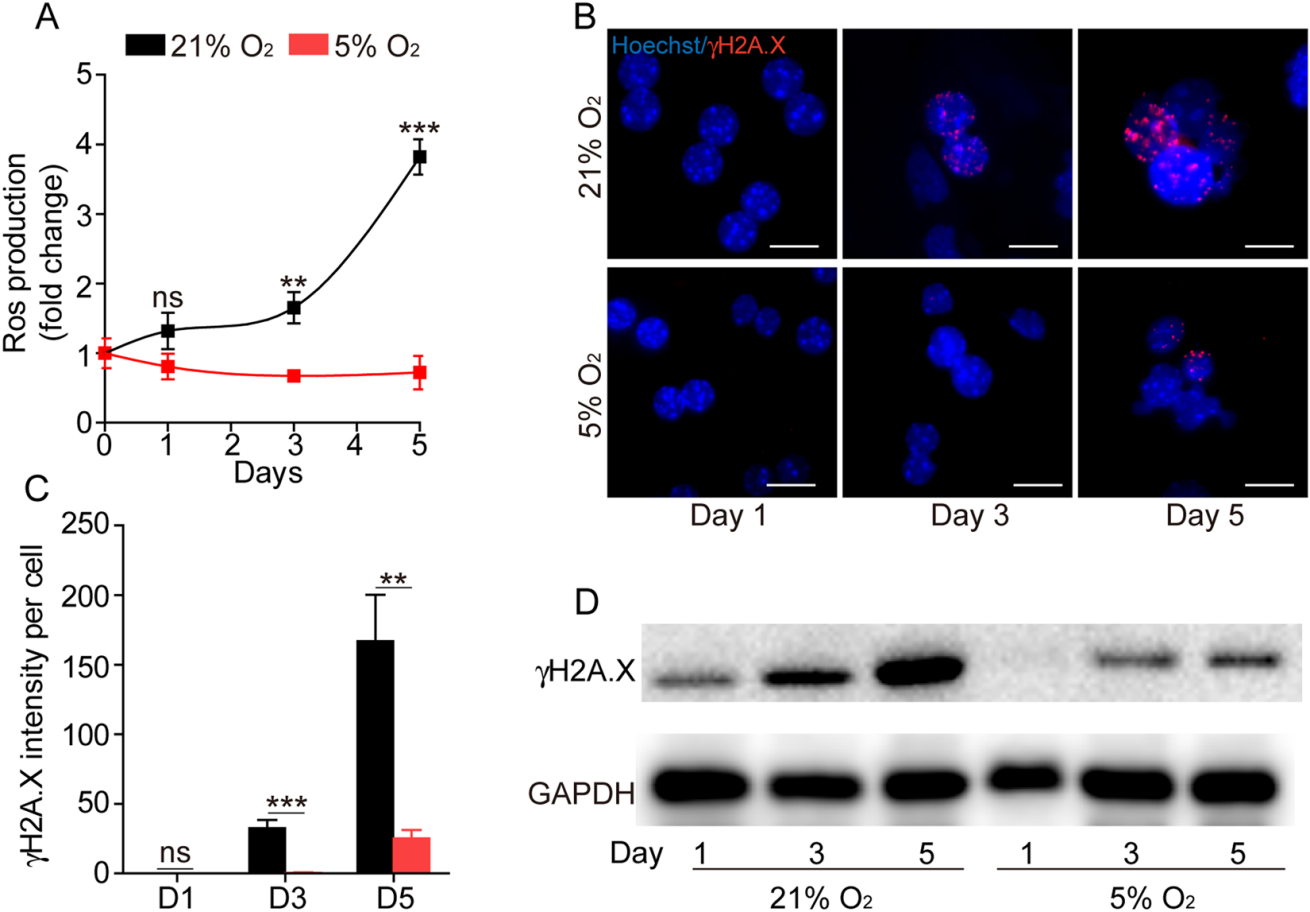
Physiological oxygen level reduces the production of ROS and DNA damage in primary hepatocytes. (**A**) Measurement of the intracellular ROS levels of primary hepatocytes cultured in 21% or 5% O_2_ at day 1, day 3 and day 5. Data are Means ± SEM (n = 6). (**B**) Immunofluorescence staining of γH2A.X in hepatocytes cultured in 21% or 5% O_2_. Nuclei were stained with Hoechst. Scale bar: 100 μm. (**C**) Statistical data of the intensity of γH2A.X staining in (**B**). Data are Means ± SEM (seven random fields) in a representative experiment. (**D**) Western blot analysis of γH2A.X in hepatocytes culture in 21% or 5% O_2_. GAPDH: loading control.

Previous studies have shown that oxidative stress plays an important role in inducing cytotoxicity and genotoxicity in human hepatoma (HepG2)^26^ as well as promoting DNA damage and apoptosis in human hepatocyte cell line L02^27^. Furthermore, reactive oxygen species (ROS) are an important factor in inducing EMT in epithelial cells^28^. To better understand whether oxidative damage is involved in the different culture conditions, we measured the ROS generated by hepatocytes cultured in 21% or 5% O_2_. As demonstrated in Fig. 5A, the intracellular ROS continued to rise in cells cultured in 21% O_2_. In contrast, the ROS level in cells cultured in low oxygen remain unchanged. ROS generation has been shown to contribute to DNA damage^29^. To better evaluate the possible influence of ROS in the two culture conditions, we determined the DNA damage level by γH2A.X staining. Hepatocytes cultured in 21% O_2_ showed a significantly higher level of γH2A.X staining comparing to the low oxygen group (Fig. 5B and C). Western blot analysis revealed a similar result (Fig. 5D). These data demonstrate that hepatocytes cultured in low oxygen tension accumulate less ROS and less DNA damage.

### ERK1/2 and GSK-3β pathways are involved in maintaining the epithelial phenotype of hepatocytes

**Figure 6:**
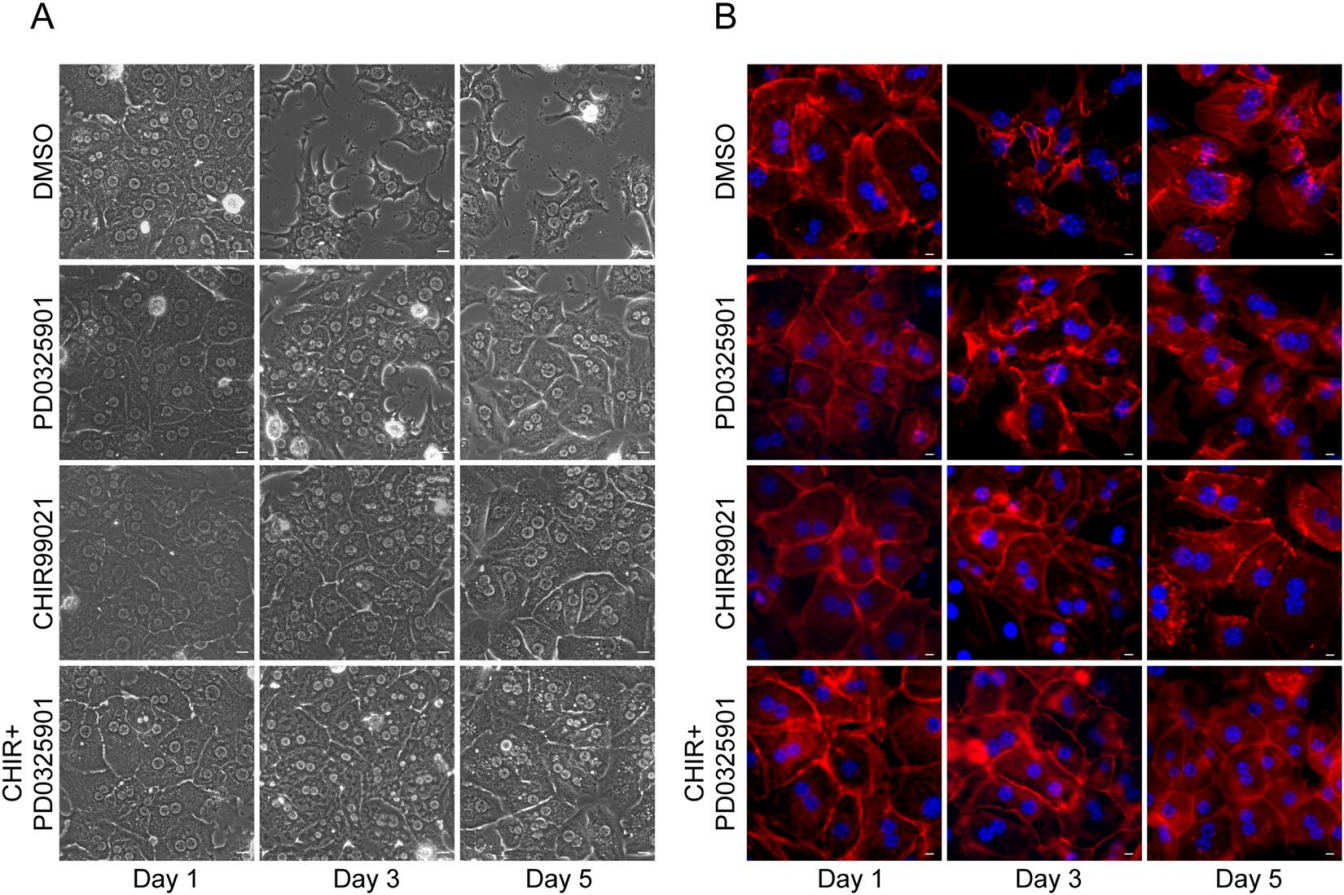
Blocking ERK1/2 and GSK-3β pathways reduce EMT in primary culture of hepatocytes. (**A**) Morphology of primary hepatocytes cultured in 21% O_2_ in the presence of ERK1/2 inhibitor (PD0325901, 10 μM) and GSK-3β inhibitor (CHIR99021, 10 μM). (**B**) Immunofluorescence staining of F-actin (red) in primary hepatocytes cultured in 21% O_2_ in the presence of PD0325901 (10 μM) and CHIR99021 (10 μM). Nuclei were stained with Hoechst.

Previous studies have shown that a vast majority of signaling pathways are involved in the regulation of EMT in various cell types. To better understand which signaling pathways may play important roles in the regulation of EMT in primary hepatocytes, we tested a number of pathway blockers, including TGF-α inhibitor Repsox and A83-01, GSK-3β inhibitor CHIR99021, notch inhibitor Compound E, ERK inhibitor PD0325901, TNF-*α* inhibitor Necrostatin-1, and HIF-1*α* inhibitor CAY10585, in primary hepatocytes cultured in 21% O_2_ condition. We found that the ERK inhibitor PD0325901 and the GSK-3β inhibitor CHIR99021 helped to maintain the epithelial phenotype of primary hepatocytes cultured in 21% O_2_ for 5 days. The combination of these two compounds provided even better results (Fig. 6). These experiments indicate that both the ERK1/2 and GSK-3β pathways are involved in the maintenance of the epithelial phenotype of hepatocytes. Thus, small molecule inhibitors targeting both pathways could be used to maintain the culture of primary hepatocytes.

## Discussion

The liver is the largest internal organ in the human body, and plays a central role in energy balance and drug metabolism. In the human body, the liver is the only organ which possesses the powerful ability to proliferate in response to partial excision, toxic injury, or infection^2^. The first liver regeneration experiment was described by Higgins and Anderson in 1931. Using a rat model, two thirds of the liver was surgically removed, and resulted in the remaining liver enlarging to original volume in approximately 1 week^30^. Unfortunately, this amazing regeneration ability is almost eliminated in *in vitro* conditions. The cultured primary hepatocytes can hardly proliferate^31^. Moreover, the current protocols for culturing of primary hepatocytes could only maintain metabolic functions for hours to a few days^32–34^. Recently, adding small molecules in culture enables 10 rounds of proliferation of human hepatocytes, but not serial passages or long-term storage of these cells^35^. Low-level expression of the human papilloma virus genes E6 and E7 could lead to up to 40 population doublings of hepatocytes cultured *in viro*, but cells with such genes modification may not be suitable for clinical-related uses^31^.

In the liver, hepatocytes reside in a microenvironment formed by extracellular matrix, blood vessels and various types of cells, making the conditions hard to mimic in culture. Hepatocytes in single layer culture rapidly lose their epithelial morphology accompanied by their specific functions such as protein synthesis (albumin, fibrinogen), bile acid production, glycogen storage, xenobiotic metabolism, etc^36^. These cells undergo a process named epithelial to mesenchymal transition (EMT)^37^ and start to accumulate actin stress fibers, display elongated “fibroblast-like” morphology, lose polarity, weaken cell-cell interaction and reduce typical hepatic functions.

*In vitro* culture for tissues and cells isolated from mammalian bodies started about a century ago. Many efforts have been put forward to maintain physiological temperature, pH, salt concentration, nutrient and necessary cell factors in the culture. Until now, in most routine cell cultures, the oxygen concentration in the medium was still simply the result of equilibration with the oxygen in air. However, in the body, many cells and tissues reside in environments with lower partial pressure of oxygen^38^. *p*O_2_ in arterial blood is 95 mm Hg, and in normal tissues it ranges from 50 to 5 mm Hg (7–0.7%), which are considerably lower than *p*O_2_ in air (150 mm Hg, 21%).

The effect of low oxygen tension in *in vitro* culture has been mostly studied in stem cell research. Low *p*O_2_ has been reported to be beneficial in maintaining the pluripotency of human embryonic stem cells (hESCs)^39^ and the generation of murine and human induced pluripotent stem cells (iPSCs)^40, 41^. EMT plays important roles in pluripotent stem cell differentiation. Low oxygen is believed to block the EMT process and therefore helps to maintain the pluripotency of ESCs^39^. Recently, a study showed that low oxygen might have direct effects on heart regeneration in adult mice *in vivo*. Adult mice maintained in 7% oxygen had less reactive oxygen species and oxidative DNA damage, and could reactivate cardiomyocyte mitosis. Exposure to low oxygen 1 week after the induction of myocardial infarction induced a robust regenerative response in adult mice, with reduced myocardial fibrosis and improved left ventricular systolic function^42^. Myocardial fibrosis after injury is also an EMT process^42^. Low oxygen may reduce myocardial fibrosis and enhance cardiomyocyte regeneration by blocking the EMT process.

Since EMT is also involved in dedifferentiation and loss of functions of hepatocytes cultured *in vitro*, it is reasonable to hypothesize that low oxygen may help to maintain hepatocyte functions in culture. Indeed, our study demonstrated that physiological oxygen tension (5% O_2_) was very effective in maintaining the epithelial morphology and many of the functions, including albumin production, glycogen storage, LDL-uptake and CYP450-mediated drug metabolism of primary hepatocytes cultured *in vitro* for up to 5 days. Lowering the oxygen tension in cell culture is easily achievable, it could be combined with other methods, such as the use of small molecule cocktails and 3D culture, to maintain the proliferation and functions of primary hepatocytes *in vitro*.

## Materials and Methods

### Animals

C57BL/6 mice were obtained from shanghai Laboratory Animal Center (Chinese Academy of Sciences) and were maintained in pathogen-free conditions with standard laboratory chow and water ad libitum. All experiments were approved and conducted in accordance with the guidelines of the Animal Care Committee of Shanghai Institute of Materia Medica.

### Primary hepatocytes isolation and culture

Primary hepatocytes were isolated from 8-week old C57BL/6 mice by standard two-step collagenase perfusion method. Briefly, the liver was perfused through the inferior vena with 30 mL Perfusion buffer I (0.5 mM EGTA, 16 mM NaHCO_3_, HBSS without Ca^2+^ and Mg^2+^) and then perfused with 25 mL Perfusion buffer II (0.4 mg/mL collagenase type IV, Gibco), 10 mM HEPES, 16 mM NaHCO_3_, HBSS with 5 mM Ca^2+^ and 1.2 mM Mg^2+^). After perfusion, the liver was removed from the abdominal cavity and hepatocytes were released into the DMEM medium using sterile surgical scissors. Cell suspension was filtered through a 350 micrometer cell strainer. Hepatocytes were purified with Percoll buffer (50% Percoll (Sigma-Aldrich), 50% DMEM) at low-speed centrifugation (1,500 r.p.m, 15 min). Viability of isolated hepatocytes was around 90% as determined by Trypan blue. Primary hepatocytes were cultured in hepato-medium (DMEM/F12 (Gibco), supplemented with 10% FBS, 1 µg/mL insulin, 100 nM Dexamethasone, 10 mM nicotinamide, 2 mM L-glutamax, 0.1 mM nonessential amino acids (NEAA), 100 units/mL penicillin and 100 µg/mL streptomycin, and 10 ng/mL EGF (Sigma-Aldrich)). The primary hepatocytes were cultured at 37 °C, 5% CO_2_ and 21% O_2_ in a regular incubator, or cultured at 37 °C, 5% CO_2_ and 5% O_2_ in a Ruskinn hypoxia workstation.

### Immunofluorescence staining

Cells were fixed with 4% PFA and incubated with primary antibodies against albumin (Bethyl A90-234A), α-SMA (Sigma A2547), Cyp1a2 (Abcam ab22717) or γH2A.X (Abcam ab26350) followed by the appropriate secondary antibodies conjugated to Alexa Fluor 555 (Invitrogen A31570) or Alexa Fluor 488 (Invitrogen A11078). Images were taken with an Olympus IX51 inverted fluorescent microscope. For F-actin staining, cells were fixed with 3.7% PFA and incubated with fluorescent phallotoxins (Gibco A34055). Nuclei were stained with Hoechst (Sigma 33342).

### PCR

Total mRNA was isolated using Trizol (Invitrogen) and 1 μg RNA was used to synthesize cDNA using PrimeScript RT reagent kit (Takara) according to the manufacturer’s protocol. Real-time PCR was performed using FastStart Universal Probe Master Mix (Roche) and a Stratagene Mx3000 P thermal cycler. Primers sequences are: Cyp1a1, forward 5′-GACCCTTACAAGTATTTGGTCGT-3′, reverse 5′-GGTATCCAGAGCCAGTAACCT-3′; Cyp1a2, forward 5′AGTACATCTCCTTAGCCCCAG-3′, reverse 5′-GGTCCGGGTGGATTC TTCAG-3′; Cyp3a11, forward 5′-TGAGGCAGAAGGCAAAGAAA-3′, reverse 5′-GGTATTCCATCTCCATCACA-3′; Cyp3a41, forward 5′-AAAGCCGCCTCG ATTCTAAGC-3′, reverse 5′-ACTACATCCCGTGGTACAACC-3′; Collage1α1 forward 5′-GCTCCTCTTAGGGGCCACT-3′, reverse 5′-CCACGTCTCAC CATTGGGG-3′; N-cadherin, forward 5′-CATGGCTCCTTCCACATGAT-3′, reverse 5′-AGGGCACCAATCACATCTGC-3′; FN, forward 5′-GAAATATTT GCTGTGTCTCAGGG-3′, reverse 5′-TAAATTTGGCACTTGCATGG-3′; Vimentin, forward 5′-GCACCACCACCCACGGAATCG-3′, reverse 5′-CGGAAAGTGGAATCCTTGCA-3′; α-SMA, forward 5′-AAAATGGA GCCAGTCACATGTGG-3′, reverse 5′-CCACCGCAAATGCTTCTAAGT-3′; Snail, forward: 5′-CACACGCTGCCTTGTGTCT-3′, reverse 5′-GGTCAGCA AAAGCACGGTT-3′; Slug, forward 5′-GAACAGTTGAGGGGCTACAC-3′, reverse 5′-GGTGAGGATCTCTGGTTTTGGTA-3′; GAPDH, forward 5′-TGGTC AAGAAACATTTCAACGCC-3′, reverse 5′-GGTCATGAGCCCTTCCAC AATG-3′.

### Western blot

Cells were lysed, sonicated and boiled at 95–100 °C for 5 min in sample buffer (50 mM Tris-HCl, 2% w/v SDS, 10% glycerol, 1% β-mercaptoethanol, 0.01% bromophenyl blue (pH 6.8)). Cell lysates were separated on SDS-PAGE and transferred to polyvinylidene difluoride membranes. The membranes were first incubated with blocking buffer (TBS with 0.05% Tween 20 and 5% non-fat milk) for 1 h at room temperature and then incubated overnight at 4 °C in buffer containing antibodies against GAPDH (Cell signaling 14C10), N-cadherin (Sigma ab76057), γH2A.X (Abcam ab26350) or α-SMA (Sigma A2547). The membranes were washed three times and then incubated with goat anti-rabbit IgG HRP (Abmart M21002) or goat anti-mouse IgG HRP (Abmart M21001) for 1 h. After washing, immunostaining was visualized using Western Lightning Ultra (Perkin Elmer) and ChemiDoc imaging system (Bio-Rad).

### PAS stain, Dil-ac-LDL and Albumin ELISA assay

To analyze the glucose storage ability of hepatocytes, cells cultured in 21% O_2_ or 5% O_2_ conditions were fixed by 4% PFA and staining with periodic acid-Schiff kit (PAS, Sigma) according to the manufacturer’s instructions. To analyze LDL-uptake ability, hepatocytes cultured in 21% O_2_ or 5% O_2_ conditions were cultured in DMEM/F12 containing DiI-Ac-LDL (10 µg/mL) for 4 h at 37 °C.Nuclei were stained by Hoechst and images were captured with an Olympus IX71 inverted fluorescent microscope. To measure albumin secretion, primary hepatocytes cultured in 21% O_2_ or 5% O_2_ conditions for 1, 3, or 5 days were transferred into fresh medium. Culture supernatant was collected 24 hr. after medium change. The amount of albumin in supernatant was determined by the mouse albumin ELISA Kit (Sigma) according to manufacturer’s instructions.

### CYP metabolism assay

For the measure of CYP enzyme activities, primary hepatocytes were cultured in 21% O_2_ or 5% O_2_ conditions and pretreated with 10 μM 3-methylcholanthrene for 48 h to induce the expression of Cyp1a1 and Cyp1a2. Cells were then dissociated and resuspended at a density of 1 million cells/mL in hepato-medium containing 200 nM phenacetin. At 0, 60, 120, 240 and 480 min time points, pipette-mix the incubate and 20 μL of the sample was transferred to a “Quenching” tubes containing 180 μL 100% acetonitrile followed by pipette-mixing. Then the sample was frozen at −80 °C until analyses. After collection of all samples, the supernatants were collected by centrifuged the mixture at 12000 g for 10 minutes and the concentration of the remained phenacetin in the supernatants were measured by LC-MS/MS system (Waters UPLC I-Class and Waters Xevo TQ-S mass spectrometer). All compounds were also purchased from Sigma-Aldrich and used as standard samples.

### Measurement of intracellular ROS production

The production of intracellular ROS was measured using the ROS detection reagents (Invitrogen) according to the manufacture’s instructions. In brief, primary hepatocytes were plated into 96-well plate and cultured in 21% O_2_ or 5% O_2_ conditions. At day 1, day 3, and day 5, the cells were washed twice with PBS and supplied with phenol red-free DMEM containing 10 μM DCFH-DA dye and then incubate for 30 min at 37 °C in dark. Cells were then washed with PBS for three times and the DCF fluorescence intensity was measured using microplate fluorescence reader (excitation wavelength: 488 nm and emission wavelength: 530 nm).

### Statistical Analyses

Values are reported as the means ± SEM. P-values were calculated by Student’s t-test, P < 0.05 was considered statistically significant. All graphs were plotted with GraphPad Prism software.
